# Magnetically Recovered Co and Co@Pt Catalysts Prepared by Galvanic Replacement on Aluminum Powder for Hydrolysis of Sodium Borohydride

**DOI:** 10.3390/ma15093010

**Published:** 2022-04-21

**Authors:** Anna M. Ozerova, Anastasia A. Skobelkina, Valentina I. Simagina, Oksana V. Komova, Igor P. Prosvirin, Olga A. Bulavchenko, Inna L. Lipatnikova, Olga V. Netskina

**Affiliations:** Boreskov Institute of Catalysis SB RAS, Lavrentiev Ave. 5, 630090 Novosibirsk, Russia; skobelkina19@gmail.com (A.A.S.); simagina@catalysis.ru (V.I.S.); prosvirin@catalysis.ru (I.P.P.); obulavchenko@catalysis.ru (O.A.B.); lil@catalysis.ru (I.L.L.); netskina@catalysis.ru (O.V.N.)

**Keywords:** cobalt-based catalyst, Co@Pt catalysts, galvanic replacement, NaBH_4_ hydrolysis, cyclic stability

## Abstract

Magnetically recovered Co and Co@Pt catalysts for H_2_ generation during NaBH_4_ hydrolysis were successfully synthesized by optimizing the conditions of galvanic replacement method. Commercial aluminum particles with an average size of 80 µm were used as a template for the synthesis of hollow shells of metallic cobalt. Prepared Co^0^ was also subjected to galvanic replacement reaction to deposit a Pt layer. X-ray diffraction analysis, X-ray photoelectron spectroscopy, scanning electron microscopy, and elemental analysis were used to investigate catalysts at each stage of their synthesis and after catalytic tests. It was established that Co^0^ hollow microshells show a high hydrogen-generation rate of 1560 mL·min^−1^·g_cat_^−1^ at 40 °C, comparable to that of many magnetic cobalt nanocatalysts. The modification of their surface by platinum (up to 19 at% Pt) linearly increases the catalytic activity up to 5.2 times. The catalysts prepared by the galvanic replacement method are highly stable during cycling. Thus, after recycling and washing off the resulting borate layer, the Co@Pt catalyst with a minimum Pt loading (0.2 at%) exhibits an increase in activity of 34% compared to the initial value. The study shows the activation of the catalyst in the reaction medium with the formation of cobalt–boron-containing active phases.

## 1. Introduction

The growing market for mobile devices and unmanned aerial vehicles stimulates the development of small-sized energy sources based on fuel cells. For their operation, it is necessary to develop chemicals-based systems for compact storage and generation of hydrogen. They should provide a high yield of hydrogen per unit weight or volume without additional heating. Among them, sodium borohydride (NaBH_4_) is of particular interest due to its high hydrogen content (10.8 wt%), commercial availability, high solubility in water (14.5 mol/L at 25 °C), inflammability, and safety in practical use [1,2]. Hydrogen evolution from NaBH_4_ may be carried out by its solid-state thermal decomposition [3,4], by its catalytic alcoholysis [5], by acid [1,6] or catalytic [7,8] hydrolysis of its solution, or by a limited supply of water to a solid hydride–catalyst composite [9,10]. Catalytic hydrolysis of NaBH_4_ (1) is the most studied and is described in a series of review articles [1,6,11,12,13,14,15,16,17]. On its basis, small-scale hydrogen generators are now being developed [18,19,20,21], including those for unmanned aerial vehicles [20,22,23].

NaBH_4_ + 4H_2_O → NaB(OH)_4_ + 4H_2_↑ ΔH_298K_ = 299 kJ/mol (1)

Among the catalysts of this process, special attention is given to cobalt, which combines high activity, abundance, and acceptable prices [1,6,11,12]. In addition, the magnetic properties of these catalysts make it easy to control the production of hydrogen by removing and reintroducing it into the reaction medium by applying an external magnetic field. Nanosized Co_x_B-based catalysts formed from different cobalt compounds under action of NaBH_4_ are the most investigated [1,6,11,12]. It is known that the activity of this phase is higher than that of metallic cobalt nanoparticles [24,25], but their magnetization is lower than those of Co^0^ [26,27]. It is also known that Co_x_B and Co^0^ nanoparticles are chemically unstable. They oxidized easily in air [28], aqueous medium [29], and an aggressive alkaline NaBH_4_ hydrolysis medium [9,30]. This is one of the reasons for their deactivation [6]. Obviously, as the contribution of the magnetic non-oxidized component decreases during oxidation, magnetic properties decrease as well.

In the literature (Table S1), there are two approaches to the synthesis of magnetic catalysts for the hydrolysis of NaBH_4_. In the first one, high concentrations of Co nanoparticles are stabilized in a non-magnetic support matrix, providing both catalytic and magnetic characteristics [31,32,33,34]. Therefore, 13.9 wt% [31] and 69.4 wt% [32] of cobalt were loaded into the cationic cryogel and the cation-exchange resin, respectively. Up to 46 wt% of Co was supported by porous spherical SiO_2_ [33]. Then, 78.1 wt% of Co was distributed on reduced graphene oxide nanosheets, which not only prevented the agglomeration of Co nanoparticles but also promoted momentum transfer in the external magnetic field [34].

Another more common method is the use of magnetic supports based on iron oxides [35,36,37,38,39,40,41,42,43,44,45] (Table S1). Fe_3_O_4_ is the most frequently used [35,36,37,38,39,40,41]. In addition, this support has some catalytic activity due to the presence of acidic and basic sites on the surface [40]. Other iron compounds are also applied, including magnetic Fe_2_O_3_ [42], CuFe_2_O_4_ [43], NiFe_2_O_4_ [44], and CoFe_2_O_4_ [45]. However, Co nanoparticles supported on bare Fe_3_O_4_ showed decreased catalytic activity [36,37,39] resulting from the involvement of iron in the redox processes [36,40]. The destruction of Fe_3_O_4_ [40] and NiFe_2_O_4_ [44] was also shown during reusability tests of NaBH_4_ hydrolysis. To protect the support surface, a coating of Fe_3_O_4_ particles by an inert shell of amorphous [36,37] or activated carbon [39], bentonite [38], or clinoptiolite zeolite [38] was proposed. This also enhanced the catalytic properties.

Despite the intensive research in this area, the use of magnetic metals as support for NaBH_4_ hydrolysis catalysts is largely unexplored. However, some articles show promising results. For example, a magnetic Fe@Co catalyst with a core-shell structure was synthesized by successive reduction of Fe^3+^ and Co^2+^ salts with a NaBH_4_ solution [46] (Table S1). In this case, both the core (Fe) and the shell (Co) have magnetic properties.

The galvanic replacement method is one of the promising methods for the synthesis of metallic cobalt particles that allows for size control [47,48]. In the traditional variant, this method is based on a redox reaction:

M_1_*^n+^* + *n*/*z*M_2_ → M_1_ + *n*/*z*M_2_*^z+^*, (2)

where M_1_ and M_2_ are different metals; M_1_*^n+^* and M_2_*^z+^* are corresponding ions. There is no need for an external current source or an additional reducing agent. The driving force for the galvanic replacement is the differences in the standard redox potentials of deposited (M_1_) and sacrificial (M_2_) metals. As a result, M_2_ is dissolved (completely or partially) as M_2_*^z+^* ions, and M_1_ is deposited in metallic form. It is believed that the replacement reaction starts at defects in the M_2_ crystal structure with a high surface energy (point defects, steps, etc.) [49]. During the process, M_1_ atoms are plated onto the entire surface of the M_2_ template particles, forming a shell. It has a porous structure due to the continuous diffusion of M_1_*^n+^* and M_2_*^z+^*. By varying the composition and concentration of the replacement solution as well as the size and shape of sacrificial metal particles, it is possible to control the conversion degree of M_2_, the thickness of the M_1_ layer, the geometry, morphology, and phase composition of the resulting samples [47,48,50]. In addition, multicomponent systems can be formed by the layer-by-layer deposition of various metals.

There are practically no studies in the literature on the preparation of catalysts for the hydrolysis of NaBH_4_ by the galvanic replacement method [25,51]. In these works, aqueous replacement solutions were used, and variations of the experimental details were practically not discussed. Hence, active hollow Ni and Co nanoparticles were synthesized using aluminum nanoparticles as a template [25]. This method was also used to deposit Au and Pt crystallites on Co fiber material, enhancing its catalytic activity [51]. However, the effect of precious metal addition on catalyst stability has not been studied and discussed.

This study is a further development of the results obtained by Cui Q. et al. (2011) [25] and Zabielaitė A. et al. (2018) [51]. The first part of the work is devoted to the synthesis of magnetically recovered cobalt-based catalysts by the galvanic replacement method using an aluminum template. By varying the synthesis parameters, namely the composition and concentration of the replacement solution, the application and duration of sonication, and Al leaching, the conditions for the formation of the most active Co catalysts for NaBH_4_ hydrolysis were identified. In the second part of the work, to improve catalytic properties, active cobalt material was additionally modified by a platinum layer. The stability of Co catalyst and Co@Pt catalysts with various platinum loadings was tested in 10 cycles of NaBH_4_ hydrolysis. The results of Co catalysts activity, the effect of Pt concentration on the activity, and deactivation process were discussed based on the study by a set of methods (elemental analysis, XRD, SEM, and XPS).

## 2. Materials and Methods

The following commercial reagents were used as received: aluminum powder—ASD-0 grade (TU 1791–007–49421776–2011, Sual-PM, Shelekhov, Russia); cobalt (III) acetylacetonate, C_15_H_21_O_6_Co—pure (TU 6-09-09-520-73, Reakhim, Moscow, Russia); acetone, CH_3_COCH_3_—analytically pure (GOST 2603-79, Baza No.1 Khimreactivov, Staraya Kupavna, Russia); hydrochloric acid, HCl—special purity 20-4 (GOST 14261-77, Sigma Tek, Khimki, Russia); sodium hydroxide, NaOH—pure (GOST 4328-77, Reakhim); chloroplatinic (IV) acid hexahydrate, H_2_PtCl_6_·6H_2_O—38.01 wt% of Pt (TU 2612-034-00205067-2003, Aurat, Moscow, Russia); sodium borohydride, NaBH_4_—purity of 98 wt% (CAS 16940-66-2, Chemical Line, Saint Petersburg, Russia).

### 2.1. Catalysts Preparation

Cobalt catalysts were synthesized by the galvanic replacement reaction using Al particles as a template using the method adapted from Cui Q. et al. [25]. First, 0.5 g of Al powder was degreased in acetone and etched in 1 M HCl solution (5 mL) for 10 min to remove the surface oxide layer. Then, 15 mL of a 0.23 M solution of C_15_H_21_O_6_Co in ethanol was added to the Al suspension. The Co:Al molar ratio was 0.2:1. The reaction was carried out in an ultrasonic bath (Sapfir, Moscow, Russia) at 60 °C and 100 W for 2 h. After the completion of the reaction (stopping the formation of gas bubbles), the resulting sample was separated from the reaction medium with a magnet, washed 5 times with distilled water and 3 times with acetone, and evacuated for 2 h at room temperature. The sample was denoted as Co(Al). The non-magnetic residual product was air dried and investigated by X-ray diffraction analysis.

To remove the residue of Al, the Co(Al) sample was treated with a 2.5 M NaOH solution for 2 h without sonication. Then, it was washed 5–10 times with distilled water to neutral pH and 3 times with acetone and evacuated for 2 h at room temperature. The sample was denoted as Co(Al)NaOH.

Co@Pt catalysts were also synthesized by the galvanic replacement method. For this, 0.1 g of the Co(Al)NaOH sample was mixed with 10 mL of an aqueous solution of H_2_PtCl_6_. The concentration of the H_2_PtCl_6_ solution was calculated from the desired Pt:Co molar ratio. The reaction was carried out in an ultrasonic bath at 35 °C and 100 W for 10 min. The resulting sample was separated from the reaction medium with a magnet, washed 5 times with distilled water and 3 times with acetone, and evacuated for 2 h at room temperature. The samples obtained in this way were denoted as Pt_x_Co_100−x_, where x = 0.2, 0.25, 2.5, 8, 19, and 55 at%.

### 2.2. Catalytic Tests in NaBH_4_ Hydrolysis

The reaction was carried out at 40 °C in a glass temperature-controlled internal mixing reactor equipped with a magnetic stirrer at an 800 rpm stirring rate. A freshly prepared aqueous solution (0.04 g in 10 mL) of NaBH_4_ was placed into the heated reactor with magneton. The catalyst (0.0117 g) was added, and the volume of generated hydrogen was measured with a 100 mL gas burette with a resolution of 0.2 mL. If noted, the experiment was carried out without stirring or with magnetic self-stirring without using a magneton. The volume of hydrogen generated was corrected to N.T.P. (20 °C; 1 atm) based on three repeated experiments under the same conditions. The experimental uncertainty was less than 2%.

Cyclic stability tests of the catalysts in the hydrolysis of NaBH_4_ were carried out at 40 °C. After completion of the hydrolysis of the first portion of hydride (0.04 g dissolved in 10 mL of distilled water), the next portion of solid NaBH_4_ (0.04 g) was placed into the reactor, stirring was turned on (800 rpm), the system was sealed, and H_2_ measurement was continued. After the 10th cycle of the reaction, the catalyst was separated from the reaction medium, washed with distilled water and acetone, and evacuated for 2 h at room temperature.

The hydrogen generation rate (W^50^) was calculated as

(3) $$W^{50}=\frac{V_{H_{2}}^{0}}{t_{1/2}\cdot m_{\mathrm{cat}}},$$

where W^50^ is the hydrogen generation rate (mL·min^−1^·g_cat_^−1^); $V_{H_{2}}^{0}$ is the volume of hydrogen produced during time t_1/2_ (mL); t_1/2_ is the time required for 50% conversion (min); and m_cat_ is the mass of the catalyst (g).

### 2.3. Catalysts Characterization

The contents of Co, Al, Pt, and B were determined by inductively coupled plasma atomic emission spectrometry on an Optima 4300 DV instrument (Perkin Elmer, Shelton, CT, USA). The relative measurement error was ±10% when the determined element concentration was less than 5 wt%, ±4% when the determined element concentration was between 5 and 10 wt%, ±3% when the determined element concentration was between 10 and 50 wt%, and ±1% when the determined element concentration was greater than 50 wt%.

The X-ray diffraction analysis was performed on a D8 Advance diffractometer (Bruker AXS GmbH, Karlsruhe, Germany) in the range of angles 5–80° with a step of 2θ = 0.05° and a time of accumulation of 5 s for each point using a Lynxeye linear detector. Cu K_α_ radiation (λ = 1.5418 Å) was used. The average coherent scattering regions (CSR) were determined using the Scherrer formula from the following reflections: 002 for Co and 111 for Pt and Al. The computation error of the CSR was 10%. The phases were identified using the following data: Co (hcp, PDF card 05-727), Al (PDF card 04-0787), Pt (PDF card 04-0802), and Al(acac)_3_ (PDF card 42-1746).

The surface morphology of the samples was examined on a JSM-6460 LV (Jeol, Akishima, Japan) scanning electron microscope (SEM). It was equipped with an INCA Energy-350 (Oxford Instruments, Oxford, UK) energy-dispersive X-ray spectrometer to reveal the chemical composition of the subsurface layer. The particle size was determined using the ImageJ program (http://rsb.info.nih.gov/ij/, accessed on 18 April 2022).

The X-ray photoelectron spectroscopy (XPS) spectra were taken with a SPECS photoelectron spectrometer (Germany) using a PHOIBOS-150-MCD-9 hemispheric analyzer and a FOCUS-500 monochromator (Al K_α_, hν = 1486.74 eV, 150 W). The binding energy (BE) scale of the spectrometer was pre-calibrated using the Au 4f_7/2_ (84.0 eV) and Cu 2p_3/2_ (932.6 eV) core level peaks. The binding energies were determined with an accuracy of ±0.1 eV. The samples were applied onto conducting Scotch tape and studied without pretreatment. The charge of the sample was taken into account using C 1s lines (284.8 eV). Analysis of the individual spectra of the elements allowed us to determine their electronic structure and to calculate the atomic concentration ratios of elements on the sample surface taking into account the element sensitivity coefficients [52].

## 3. Results and Discussion

### 3.1. Synthesis and Investigation of Cobalt-Based Catalysts

Cobalt catalysts were synthesized by the galvanic replacement reaction using Al particles as template. Depending on the oxidation state of cobalt in the replacement solution, the process is based on the following chemical reactions:

3Co^2+^ + 2Al^0^ → 3Co^0^ + 2Al^3+^ or (4)

Co^3+^ + Al^0^ → Co^0^ + Al^3+^, (5)

which are possible because the standard reduction potentials of Co^2+^/Co (−0.28 V vs. the standard hydrogen electrode (SHE)) and Co^3+^/Co (+0.45 V vs. SHE) are bigger than that of Al^3+^/Al (−1.66 V vs. SHE) [53].

**Figure 1:**
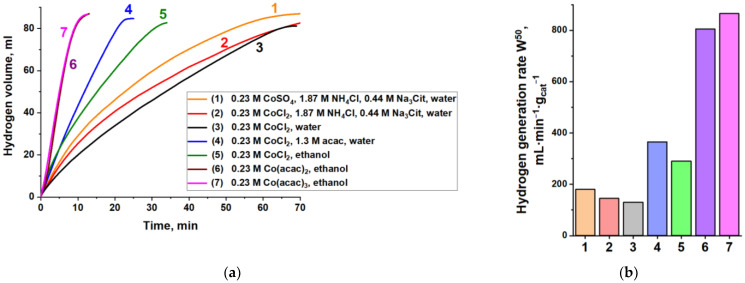
The effect of the composition of the replacement solution in the synthesis of cobalt catalysts on their activity in the hydrolysis of NaBH_4_. T = 40 °C. (**a**) Kinetic curves of hydrogen evolution over time; (**b**) hydrogen-generation rate W^50^. The synthesis of the catalysts is described in the Supplementary Materials.

In this work, the synthesis technique was adapted from Cui Q. et al. [25]. The differences are the use of another source of aluminum and the application of sonication to improve the heat and mass transfer and quicken the reaction rate. Furthermore, the nature of the cobalt salt (CoSO_4_, CoCl_2_, Co(acac)_2_, Co(acac)_3_), solvent (water, ethanol), and complexing agent (sodium citrate, acetylacetone) in the replacement solution were varied (Figure 1). The preparation methods for all catalysts are presented in the Supplementary Materials. Our results confirm that using water replacement solutions requires the addition of complexing and buffering agents (solutions No. 1, 2, 3). It is compositions No. 1 and 2 that were used in the work of Cui Q. et al. [25]. Wherein, acetylacetone as a complexing agent was found to be more effective than standard sodium citrate with NH_4_Cl (No. 2, 4). On the other hand, the literature shows that the non-aqueous medium for galvanic replacement has a significant effect on the morphology and properties of the deposited layers [54,55]. Indeed, using ethanol instead of water in the replacement solution resulted in the formation of a more active catalyst (No. 3, 5). Taking into account the obtained results on the influence of ethanol and acetylacetone, a new composition of the replacement solution was proposed (No. 6, 7). It was shown that cobalt acetylacetonate as a cobalt precursor and ethanol as a solvent are more promising. However, our results show that the difference in the reduction potentials of Co^2+^ and Co^3+^ in acetylacetonates does not have a significant effect on the hydrogen-generation rate. It is 805 mL·min^−1^·g_cat_^−1^ in the case of Co(acac)_2_ and 865 mL·min^−1^·g_cat_^−1^ in the case of Co(acac)_3_. Moreover, when cobalt acetyacetonates are used, XRD analysis shows that the bulk monoclinic structure of aluminium acetylacetonate Al(acac)_3_ (PDF card 42-1746) is the main non-magnetic residual product (Figure S1). Aluminum acetylacetonate is a useful organometallic source of aluminum that is soluble in organic solvents. It is used, for example, as a precursor for the synthesis of aluminum oxide, Al_2_O_3_ [56], including in the form of thin films by chemical vapor deposition [57,58] or nanoparticles [58], as well as several polymers [59].

Note that in the work of Cui Q. et al. [25], the synthesis time was more than 96 h without sonication and heating the reaction mixture with a volume of 15 mL. According to our results, under sonication and heating, the process was completed in 2 h. However, the proportional increase of the loading of reagent by two and eight times requires the increase of sonication duration by two and four times, respectively (Figure S2). Stopping the process before the chemical reaction is completed (gas-formation stops) leads to the formation of low-active systems, probably due to the low deposition of catalytically active cobalt. Sonication for too long is also impractical since it leads to an increase in energy consumption as well as a slight decrease in the activity of the resulting catalysts (Figure S2). An increase in the concentration of cobalt in the replacement solution is also not justified since it does not lead to an increase in activity (Figure S3) and increases the amount of unreacted cobalt. Thus, the yield of the catalyst at a molar ratio of Co:Al = 0.2:1 is 87%, at Co:Al = 0.4:1—37%, and at Co:Al = 0.6:1—only 9%.

Thus, it was found that using ethanol Co(acac)_3_ solution with the molar ratio Co:Al = 0.2:1 is optimal for the formation of the highly active Co(Al) catalyst. Synthesis should be carried out using sonication, the time of which is determined by the volume of the reaction mixture. For 0.5 g of Al powder and 20 mL of solution, the reaction is completed in 2 h at 60 °C.

**Figure 2:**
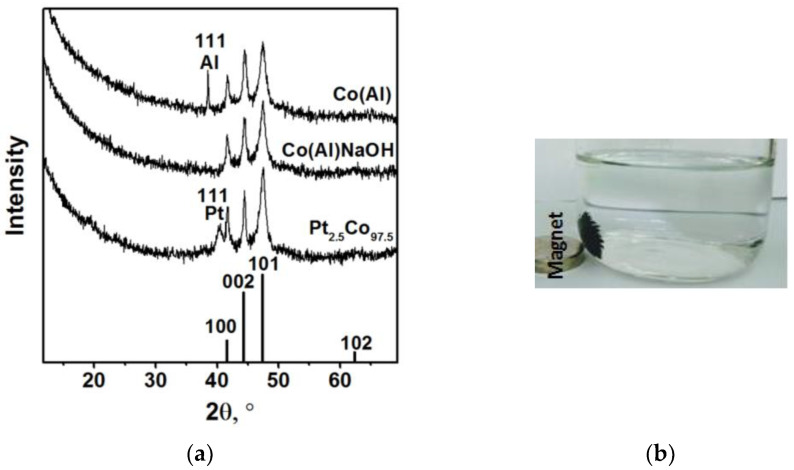
(**a**) XRD patterns of Co(Al), Co(Al)NaOH, and Pt_2.5_Co_97.5_ and bar chart of the XRD peaks for metallic hcp Co (PDF card 05-727). Al—metallic aluminum (PDF card 04-0787); Pt—metallic platinum (PDF card 04-0802). (**b**) Photographic image of Co(Al) in the presence of an external magnet showing its magnetic properties.

The catalyst obtained under these conditions, denoted as Co(Al), was investigated by physico-chemical methods. According to the elemental analysis, it contains 57.8 wt% of Co and 10.5 wt% of Al. The low concentrations of both Co and Al are likely due to the presence of amorphous Al(OH)_3_·nH_2_O forming as a product of aluminum hydrolysis. Indeed, XRD does not reveal any oxidized phases but reveals the crystalline phases of Al^0^ with a CSR value of 130 nm and Co^0^ with a CSR value of 15 nm (Figure 2a, Table 1). Note that the value of CSR of the initial Al is >150 nm (Table 1). A comparison of the Co(Al) XRD pattern with the bar chart of the XRD peaks for hcp cobalt (PDF card 05-727) shows the absence of 102 reflection. In addition, the main 101 reflection at 2θ = 47.5° is significantly broadened, and the observed peaks at 2θ values of 41.6 and 44.5° have different widths (Figure 2a). These diffraction features indicate the presence of stacking faults in Co^0^ [60,61]. It is for this reason that, in the case of metallic Co, the CSR was calculated using the 002 line since the influence of stacking fault on the 002 reflection is minimal.

**Figure 3:**
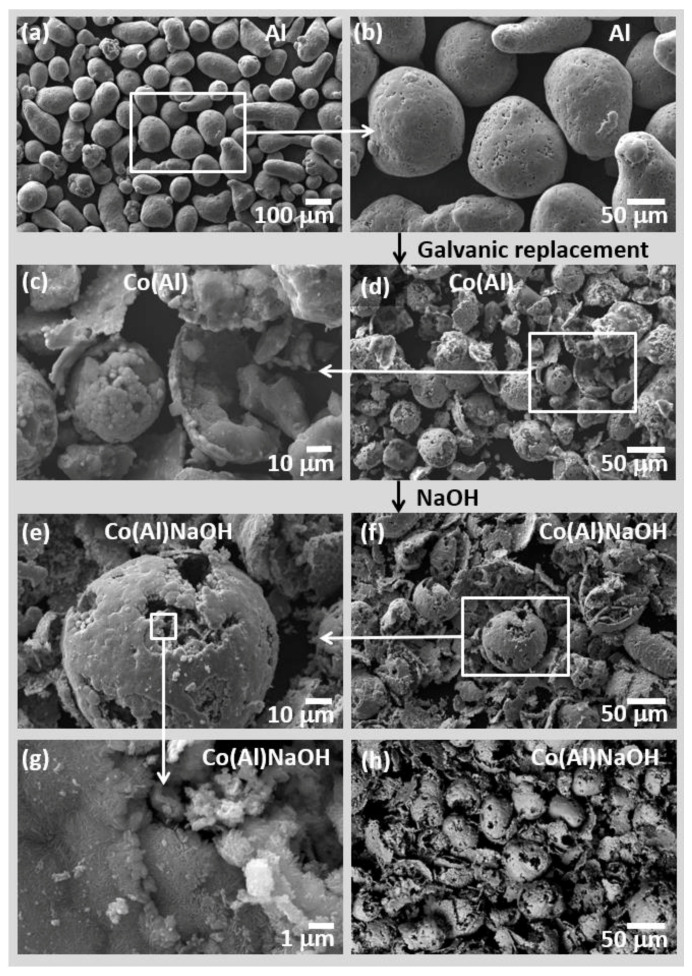
SEM images of initial Al powder (**a**,**b**) and synthesized Co(Al) (**c**,**d**) and Co(Al)NaOH (**e**–**h**).

The morphology of the synthesized Co(Al) catalyst was studied by SEM (Figure 3). The micrographs of the initial Al sample (Figure 3a,b) indicate that it consists of polydisperse, nonporous particles with spherical and ellipsoidal shapes and a smooth surface. The particle size is 35–125 μm; the average particle size is 80 ± 18 μm. After the galvanic replacement reaction in the Co(acac)_3_ solution (Figure 3c,d), the average particle size decreases (45 ± 13 μm). It is consistent with XRD data on a decrease in the CSR size of Al (Table 1). Indeed, the atomic radius of Al is 1.43 Å, and the lattice constant for Al with an fcc crystal structure is 4.046 Å, while in the case of Co, the atomic radius is 1.25 Å, and the lattice constants for Co with an hcp crystal structure are 2.507 and 4.070 Å. Additionally, the reduced unit cell volume decreases from 16.6 to 11.1 Å^3^ when aluminum is replaced by cobalt. As a result of these differences, mismatch strains obviously form. Figure 3c shows that the surface of the Co(Al) sample is covered with a rough layer of cobalt particles of 2–3 μm in size, closely adjacent to each other. The destruction of a small part of the Al particles is observed. The thickness of the cobalt shell is ~3 µm (Figure 3c).

The synthesized Co(Al) catalyst is magnetic and instantly collected on a magnet (Figure 2b). In addition to easy separation from the reaction medium, the pronounced magnetic properties of the Co(Al) catalyst make it possible to stir the NaBH_4_ hydrolysis medium under the action of an external magnetic field without using a magneton (Figure S4). Furthermore, stirring Co(Al) results in a higher hydrogen yield and a two-fold reduction in reaction time when compared to a static state. In the literature, Pd/C-dots@Fe_3_O_4_ [35] and Co@g-C_3_N_4_-rGO [34] magnetic catalysts were also used in the self-stirring mode.

**Figure 4:**
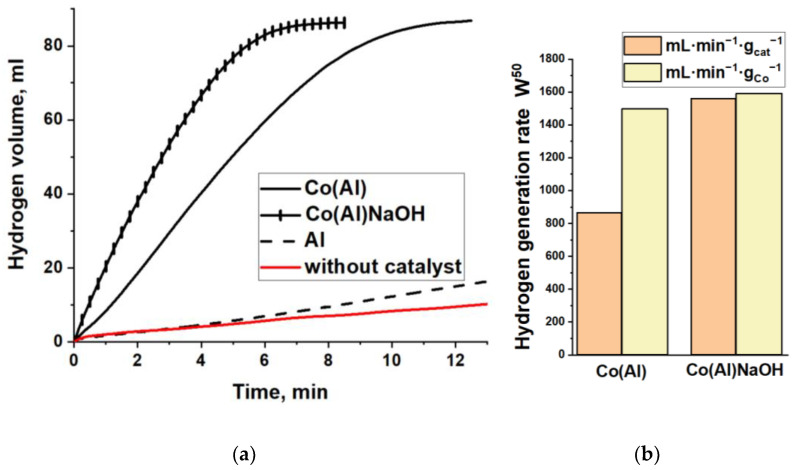
(**a**) Kinetic curves and (**b**) rate W^50^ of hydrogen evolution during the hydrolysis of NaBH_4_ without a catalyst and in the presence of Al, Co(Al), and Co(Al)NaOH. T = 40 °C.

As noted above, the Co(Al) catalyst contains 10.5 wt% of Al. Under the conditions of NaBH_4_ hydrolysis used in this work, aluminum does not contribute to the generation of hydrogen (Figure 4). The rates of H_2_ evolution upon non-catalytic hydrolysis of NaBH_4_ and upon the addition of Al to the reaction medium are low and almost coincide (Figure 4). For this reason, Al was removed from the Co(Al) sample by treatment with a 2.5 M NaOH solution for 2 h. The resulting sample was denoted as Co(Al)NaOH. In the first minutes of the reaction, intense gas evolution was observed due to the well-known chemical processes:

2Al + 2NaOH + 6H_2_O → 2Na[Al(OH)_4_] + 3H_2_↑, (6)

Al_2_O_3_ + 2NaOH + 3H_2_O → 2Na[Al(OH)_4_]. (7)

Elemental analysis confirmed that this NaOH treatment led to almost complete removal of Al from the sample. Its residual amount is 0.06 wt%. The content of cobalt increased from 57.8 to 98.1 wt%. XRD data also show that Co^0^ (hcp) is the only crystalline phase of the Co(Al)NaOH sample (Figure 2a). Simultaneously, the value of SCR of Co decreases slightly from 15 to 12 nm (Table 1). As can be seen from the SEM images (Figure 3e–h), Co(Al)NaOH is composed of hollow particles of metallic cobalt in Co(Al) particle shapes. Some of the shells were destroyed under the action of rapidly released hydrogen. The high-magnification SEM images (Figure 3g,h) demonstrate that the Co(Al)NaOH surface is covered by small, close-packed plates. This lamellar morphology is characteristic of cobalt hydroxide Co(OH)_2_ [62,63].

The study of the catalytic properties showed that the hydrogen-generation rate during the NaBH_4_ hydrolysis in the presence of cobalt hollow spheres Co(Al)NaOH is 1.8 times higher than in the presence of the initial Co(Al) sample containing aluminum (Figure 4a). However, the calculation of W^50^ per cobalt mass in the catalyst shows similar values for these samples (Figure 4b). Therefore, despite severely changing the morphology and chemical composition, NaOH treatment did not affect the activity normalized to the cobalt content. The hydrogen-generation rates obtained for Co(Al)NaOH, namely 1560 mL·min^−1^·g_cat_^−1^ at 40 °C, 920 mL·min^−1^·g_cat_^−1^ at 30 °C, and 585 mL·min^−1^·g_cat_^−1^ at 25 °C, are within the ranges of values typically obtained for cobalt catalysts (Table S1). The apparent activation energy calculated using the Langmuir–Hinshelwood model was determined to be 55.5 ± 1.9 kJ·mol^−1^ (see the Supplementary Materials, Figure S5, Table S3). This value is close to the value of 63 kJ/mol, which was determined for hollow cobalt nanoparticles of 100–200 nm in size prepared by the galvanic replacement method [25], and is also characteristic for other cobalt-based catalytic materials (Table S1). To increase the catalytic activity and stability, the synthesized hollow cobalt spheres were doped with platinum. Platinum, like other noble metals, has been shown to have superior catalytic activity [1,2,13,16].

### 3.2. Co@Pt Catalysts

The Co(Al)NaOH catalyst was used as a template for the galvanic replacement reaction with H_2_PtCl_6_ solution of various concentrations according to:

2Co + PtCl_6_^2−^ → Pt + 2Co^2+^ + 6Cl^−^. (8)

**Figure 5:**
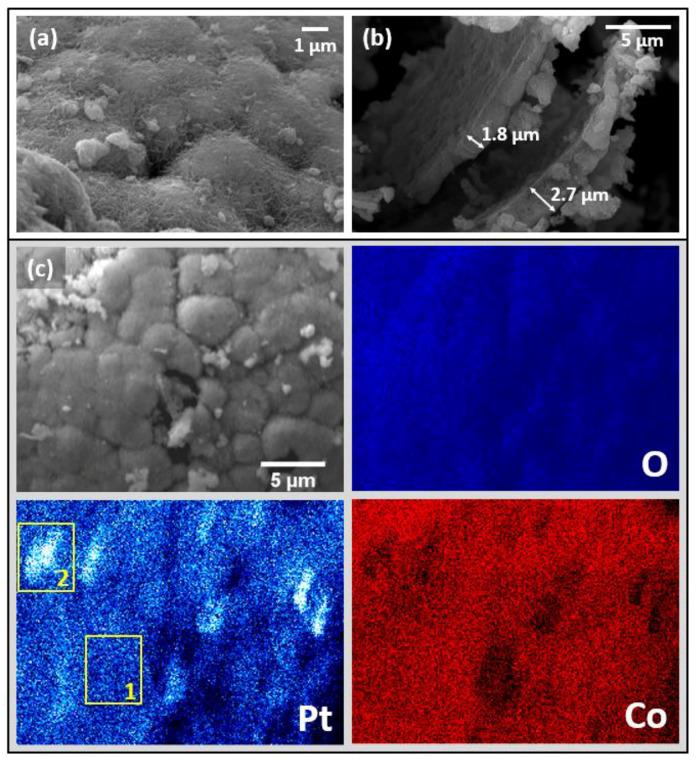
SEM (**a**,**b**) and EDX element mapping (**c**) of Pt_2.5_Co_97.5_. Rectangle 1 is an area with uniformly distributed Pt particles. Rectangle 2 is an area with elevated Pt concentrations.

The standard reduction potential of the PtCl_6_^2-^/Pt redox pair is 0.72 V vs. SHE [53], which is much higher than that of the Co^2+^/Co redox pair. So, the reaction (8) proceeds upon contacting Co particles with the H_2_PtCl_6_ solution. As a result, the compositions containing 0.59 wt% Pt (Pt_0.2_Co_99.8_), 0.82 wt% Pt (Pt_0.25_Co_99.75_), 7.82 wt% Pt (Pt_2.5_Co_97.5_), 21.6 wt% Pt (Pt_8_Co_92_), 43.7 wt% Pt (Pt_19_Co_81_), and 76.9 wt% Pt (Pt_55_Co_45_) were obtained. Note that roughly half of the initial Co(Al)NaOH hollow spheres were destroyed during the Pt deposition, most likely due to sonication (Figure 5a). Their thickness (1.5–3 μm) did not change much (Figure 5b). It should be pointed out that using the Co(Al) sample containing Al for the synthesis of Pt_x_Co_100−x_ catalysts is not justified. It is due to the spontaneous reduction of Pt on the Al surface that results in the formation of non-magnetic particles of platinum black. This reduces the yield of the target magnetic product of Pt_x_Co_100−x_.

According to XRD data, platinum in the Pt_x_Co_100−x_ samples is in the metallic state (Figure 2a, Table 1). In the case of Pt_2.5_Co_97.5_, it is evidenced by the appearance in the XRD pattern of broad peaks at 40.2° and 46.6°, corresponding to 111 and 200 reflections of Pt. The 200 peak of Pt and the 101 peak of Co overlap, and therefore, the shoulder in the region of smaller angles at the 101 Co reflection is observed (Figure 2a). The crystallite size of Pt is 10 nm (Table 1). According to SEM and EDX mapping (Figure 5), platinum is uniformly distributed on the cobalt surface, repeating its morphology. However, there are also areas with elevated Pt concentrations, which may possibly be aggregates of nanosized platinum crystallites. On the SEM image, they are observed as bright white regions (rectangle 2 on Figure 5c).

**Figure 6:**
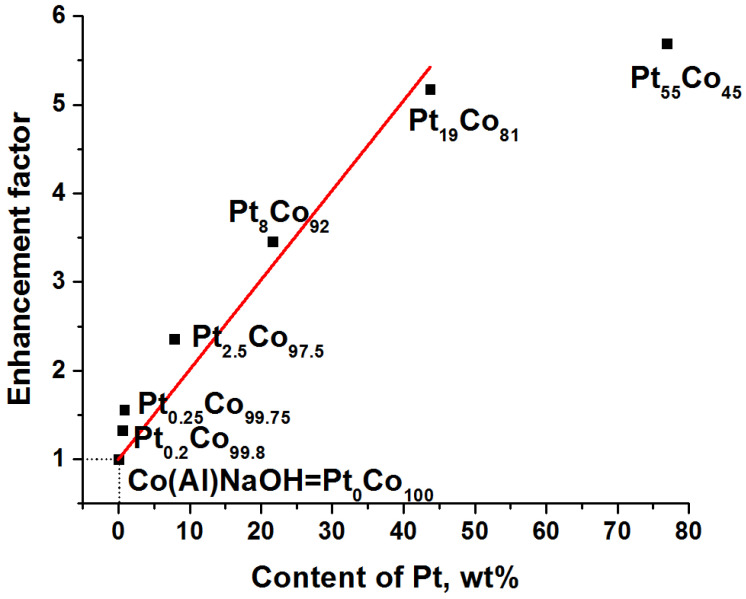
The activity enhancement factor (W^50^_PtCo_/W^50^_Co(Al)NaOH_) of Pt_x_Co_100−x_ catalysts in the NaBH_4_ hydrolysis at 40 °C depending on Pt loading (wt%).

Catalytic tests of Pt_x_Co_100−x_ in the hydrolysis of NaBH_4_ show a linear increase in activity with the addition of up to 19 at% platinum to the cobalt catalyst (Figure 6). For example, the modification of the Co(Al)NaOH catalyst with 0.2 at% Pt increases the hydrogen generation rate by 1.3 times; 0.25 at% Pt by 1.6 times; 2.5 at% Pt by 2.3 times; 8 at% Pt by 3.4 times; and 19 at% Pt by 5.2 times. However, the further increase in platinum content does not have such a significant effect. The replacement of more than half of the cobalt atoms by platinum (55 at%) leads to an increase in the activity of the catalyst only by 5.9 times.

**Figure 7:**
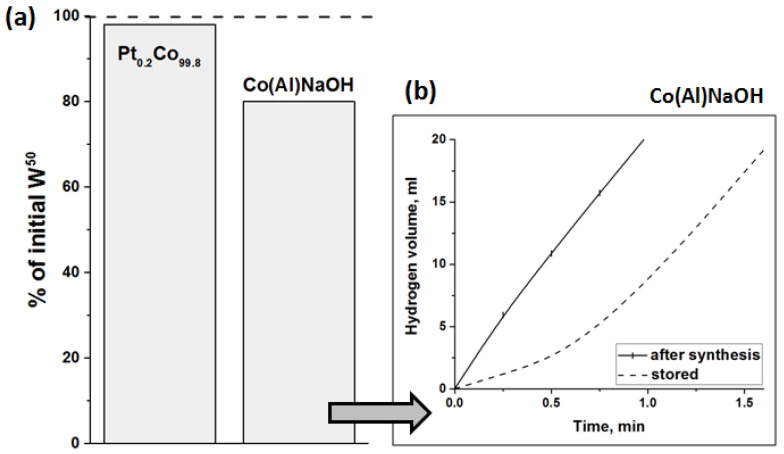
Pt_0.2_Co_99.8_ and Co(Al)NaOH storage stability. (**a**) The catalytic activity of the catalysts stored in air for 1 month, expressed as % of the initial value of W^50^, determined during testing of freshly synthesized samples. (**b**) The initial stage of the hydrogen evolution in the presence of stored and freshly synthesized Co(Al)NaOH. T = 40 °C.

Note that the addition of even a minimal amount of platinum to the cobalt catalyst also makes it more stable during storage in air (Figure 7). As shown in Figure 7b, an induction period with a low rate of hydrogen formation appears at the initial stage of the reaction over the Co(Al)NaOH sample stored for 1 month. According to previous studies [64,65,66], it is determined by the time required for the oxygen-containing cobalt compounds to become reduced in the reaction medium of NaBH_4_ hydrolysis. The average hydrogen-generation rate (W^50^) is only 80% of the W^50^ value obtained for a freshly synthesized sample. It should be mentioned that the further storage has no effect on the duration of the induction period or the activity of the Co(Al)NaOH catalyst. On the contrary, the stored Pt_0.2_Co_99.8_ catalyst exhibits 98% of its initial activity; the induction period does not appear (Figure 7). The initial Co(Al)NaOH sample appears to be oxidized in air during storage, whereas the platinum-containing Pt_0.2_Co_99.8_ sample appears to be less prone to oxidation. This effect is observed at low Pt content when not all the surfaces of cobalt are covered by a platinum layer. It can be proposed that Pt helps the rapid reduction of the surface cobalt oxide layer in the reaction medium of NaBH_4_. According to [67,68], Pt can facilitate the cleavage of the B-H bond of borohydride-anion to form active hydridic Pt-H species, which may rapidly reduce the oxidized states of cobalt. On the other hand, there may be an activation of evolved H_2_ via its dissociation on the Pt surface. According to [69,70], this resulted in the almost complete reduction of cobalt oxides in Co nanoparticles at 38 °C in an H_2_ atmosphere.

### 3.3. Catalysts Cycling Stability

Reusability is an important feature of heterogeneous catalysts since it determines their commercial applicability. In this work, the stability of the catalysts was investigated by reusing them in the NaBH_4_ hydrolysis for 10 cycles. After the end of a typical experiment (first cycle) at 40 °C, a new portion of solid NaBH_4_ was added to the reactor containing the catalyst and water solution of products.

**Figure 8:**
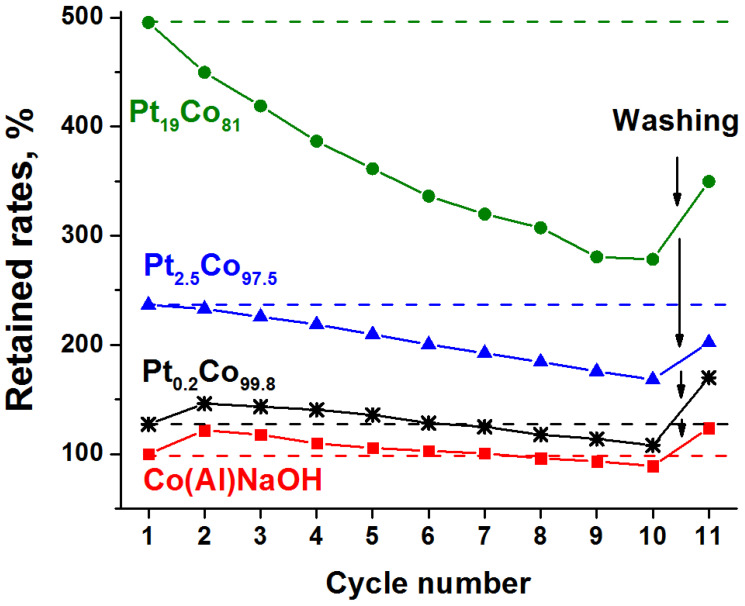
NaBH_4_ hydrolysis cycling experiments for Co(Al)NaOH, Pt_0.2_Co_99.8_, Pt_2.5_Co_97.5_, and Pt_19_Co_81_ catalysts. Retained rates ($\frac{\mathrm{current}\;W^{50}}{W_{\mathrm{Co}\left(\mathrm{Al}\right)\mathrm{NaOH}}^{50}\;\mathrm{in}\;1\mathrm{st}\;\mathrm{cycle}}\times 100\%$) as a function of cycle number.

Figure 8 shows that the change in the activity of the cobalt catalyst Co(Al)NaOH and the catalyst with minimum platinum loading Pt_0.2_Co_99.8_ is quite similar. There is an increase in the hydrogen-generation rate in the second cycle, which is characteristic of cobalt systems [30,71,72,73]. This is believed to be due to the reduction of oxidized cobalt compounds under the action of NaBH_4_ into a highly active, amorphous phase of cobalt boride Co_x_B [64,65,73]. Further, the generation of hydrogen gradually decreases. It equals the initial first cycle value in the seventh cycle, and it is 85 and 89% in the tenth cycle for Co(Al)NaOH and Pt_0.2_Co_99.8_, respectively.

Traditionally, a decrease in the activity of catalysts during the hydrolysis of NaBH_4_ is associated with the accumulation of borate anions on their surface [6]. Elemental analysis does show the presence of boron in the tested samples (Table S4). Its concentration decreased after the catalysts were thoroughly washed with water and then evacuated (Table S4). The results show complete recovery of Co(Al)NaOH activity after washing, and the activity of platinum-containing Pt_0.2_Co_99.8_ even increased by 16% compared with the second cycle (Figure 8). This growth in activity is unlikely to be due solely to the removal of the borate layer.

**Figure 9:**
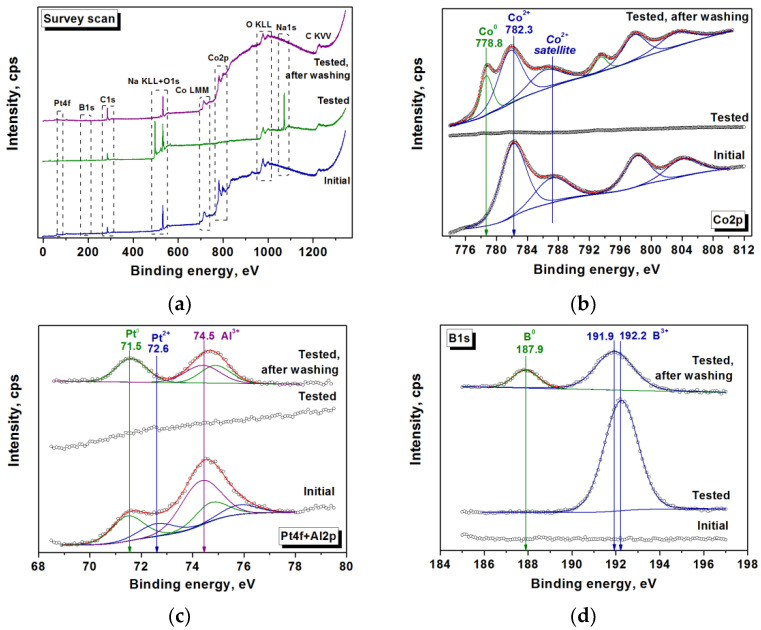
XPS data for the Pt_0.2_Co_99.8_ sample: initial and after testing in 10 cycles of the NaBH_4_ hydrolysis without and with water washing. Survey spectra (**a**), deconvolution of Co 2p spectra (**b**), Pt 4f + Al 2p spectra (**c**), and B 1s spectra (**d**).

Therefore, the surface of the Pt_0.2_Co_99.8_ catalyst was characterized by the XPS method (the analysis depth was about 5 nm). It is shown that on the surface of the initial untested sample, cobalt is in the oxidized state (Figure 9b). The peak at 782.3 eV and the satellite peak at 787.2 eV in the Co 2p_3/2_ XPS spectrum are attributed to Co^2+^ species in the oxygen environment [74,75]. The peak in the Pt 4f region has an asymmetric shape (Figure 9c). Its deconvolution is difficult due to a significant overlap between the Pt 4f and Al 2p regions. It may be stated that platinum in the catalyst is predominantly in the zero-valent metallic state Pt^0^ (peak at 71.5 eV [76]) with some contribution from Pt^2+^, most likely in the form of PtO or Pt(OH)_2_ (peak at 72.6 eV [77]).

XPS revealed that Pt and Co were not found on the surface of the Pt_0.2_Co_99.8_ catalyst after its testing in 10 cycles of NaBH_4_ hydrolysis, but B, Na, and O were present (Figure 9a, Table 2). This confirms the formation of the borate layer. Indeed, a 192.2 eV binding energy was found for B 1s (Figure 9d), which coincides with the value characteristic of B^3+^ in borate anions [78]. The presence of borate anions was also detected using FTIR spectroscopy (Figure S6). Washing the tested catalyst with water leads to a decrease in the boron concentration and the complete removal of sodium (Table 2). This indicates the removal of water-soluble sodium borates from the surface. The remaining boron exists in two states (Figure 9d). Oxidized B^3+^ with a binding energy of 191.9 eV (76%) probably corresponds to borate anions associated with Co^2+^. In addition to B^3+^, a new B 1s peak appears at the binding energy of 187.9 eV (Figure 9d), which is typical for boron in amorphous Co_x_B compounds [73,79,80,81]. In the Co 2p3/2 region, there is also a new peak at 778.8 eV (Figure 9b), which is assigned to metallic cobalt Co^0^ [73,75,79,80,82]. Therefore, on the surface of the Pt_0.2_Co_99.8_ catalyst tested in 10 cycles of NaBH_4_ hydrolysis and washed with water, 24% of the total boron is in the form of B^0^, and 29% of the total cobalt is in the form of Co^0^. All platinum is in the metallic Pt^0^ form (Figure 9c). Thus, the increase in the activity of the Pt_0.2_Co_99.8_ catalyst after long-term testing and washing can be ascribed to the in situ formation of the active Co_x_B phase under the action of NaBH_4_ solution as well as the formation of the cobalt–borate phase by the interaction of borate anions and Co^2+^. As shown earlier [65], cobalt borate is highly active in the process under study.

**Figure 10:**
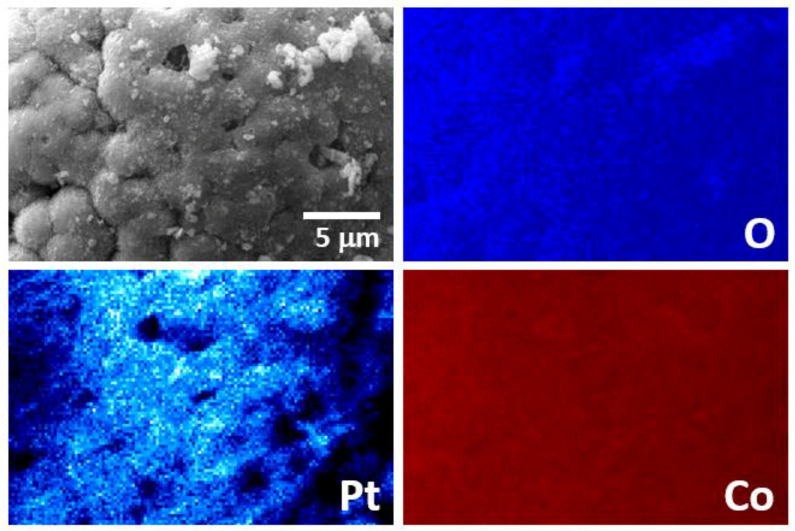
EDX element mapping of Pt_2.5_Co_97.5_ tested in 10 cycles of NaBH_4_ hydrolysis and washed.

The change in the activity of platinum–cobalt catalysts with higher platinum loadings, Pt_2.5_Co_97.5_ and Pt_19_Co_81_, differs from those of Co(Al)NaOH and Pt_0.2_Co_99.8_ (Figure 8). There is no increase in activity in the second cycle, and further deactivation is more pronounced. The increase in activity following the removal of the surface borate layer is not significant. The Pt_2.5_Co_97.5_ catalyst shows 71% of the initial hydrogen generation rate W^50^ in the tenth cycle, which after washing increases to 85%. For the Pt_19_Co_81_ catalyst, these values are only 56 and 71%, respectively (Figure 8). This difference is probably due to the little contact of cobalt with the reaction medium when a more complete coverage of the cobalt surface with platinum is realized. Hence, the in situ formation of a sufficient amount of active cobalt boride or cobalt–borate phases is difficult. Furthermore, the concentration of platinum has decreased. According to elemental analysis data (Table S4), about 21 and 58 mg of Pt were washed out of 1 g of Pt_2.5_Co_97.5_ and Pt_19_Co_81_ catalysts during 10 cycles, respectively. EDX mapping shows that the remaining part of Pt is uniformly distributed over the surface in the form of fine particles (Figure 10). It is possible that weakly bonded aggregates of platinum observed in the initial Pt_2.5_Co_97.5_ (Figure 5c) are split off in the course of catalytic experiments. The cobalt distribution in the tested Pt_2.5_Co_97.5_ (Figure 10) is as uniform as in the initial sample (Figure 5). Additionally, note that, despite the intense gas evolution, no additional destruction of catalyst spheres and shells occurs during cyclic tests (Figure S7).

## 4. Conclusions

In this work, magnetic cobalt-based catalysts for the hydrolysis of NaBH_4_ were synthesized by the galvanic replacement method using commercial aluminum particles of ~80 μm in size as a template. Our results show that Co(acac)_3_ is a more promising cobalt precursor than CoCl_2_ and CoSO_4_, and using alcohol replacement solution gives a more active Co catalyst than in the case of aqueous medium, while sonication can significantly reduce the synthesis time. According to XRD and SEM, particles formed during the galvanic replacement of Al have an average size of ~45 μm and consist of an aluminum core covered with a rough shell of metallic cobalt (hcp) about 3 μm thick. Due to magnetization, easy separation of catalyst from the reaction medium by a magnet and effective stirring of the reaction medium without a magneton under the action of an external magnetic field were observed.

It was found that the treatment of the sample with NaOH solution led to the removal of almost all aluminum and the formation of hollow particles of metallic cobalt. Its catalytic activity is 1.8 times higher than that of Al-containing particles. It is associated with an increase in the content of active cobalt in the sample. The hydrogen-generation rate in the presence of these hollow cobalt microspheres is 1560 mL·min^−1^·g_cat_^−1^ at 40 °C, 920 mL·min^−1^·g_cat_^−1^ at 30 °C, and 585 mL·min^−1^·g_cat_^−1^ at 25 °C.

In the next stage, the synthesized magnetic hollow microshells of metallic cobalt were modified with a platinum layer by their galvanic replacement in H_2_PtCl_6_ water solution. As a result, according to XRD and SEM, effective use of Pt is achieved by its uniform thin-layer distribution on the surface of the Co shells. The magnetic cobalt core provides an easy separation and recycling of the catalyst. Furthermore, platinum was shown to improve the stability of cobalt catalysts during storage. The addition of only 0.2 at% Pt increases the catalytic activity by 1.6 times. Upon further addition of up to 19 at% Pt, a linear increase in activity up to 5.2 times is observed. The addition of more Pt does not have such a significant effect.

Reusability tests show the high stability of the synthesized catalysts. According to XPS, during the NaBH_4_ hydrolysis, they undergo the following processes: (1) adsorption of sodium borates on the catalyst surface; (2) in situ activation of the part of the cobalt surface under the action of NaBH_4_ to highly active cobalt boride and probably cobalt–borate phases; and (3) partial removal of the platinum that is more pronounced for the catalysts with a high Pt concentration. The hydrogen-generation rate after 10 cycles of testing hollow Co microshells and low platinum Pt_0.2_Co_99.8_ catalyst and washing off the borate layer exceeds the initial activity. Therefore, taking into account the problem of Pt leaching and the achieved results on the catalyst activity and stability, the use of a low-concentrated Co@Pt catalyst (up to 2.5 at% of Pt) is more promising and cost-effective.

## Figures and Tables

**Table 1:** XRD analysis data for initial Al and synthesized Co(Al), Co(Al)NaOH, and Pt_2.5_Co_97.5_ catalysts.

Sample	Phase Composition	CSR (nm)
Al	Al	>150
Co(Al)	Co Al	15 130
Co(Al)NaOH	Co	12
Pt_2.5_Co_97.5_	Co Pt	30 10

**Table 2:** XPS data of surface composition of the Pt_0.2_Co_99.8_ sample: initial and after testing in 10 cycles of the NaBH_4_ hydrolysis without and with water washing.

Sample	Content, at%					
	Co	Pt	B	O	Na	Al
Initial	14.9	0.1	0	40.0	0	2.0
Tested	0.2	0	8.3	33.8	11.4	0
Tested, after washing	5.8	0.05	4.3	32.4	0	0.8

## Data Availability

Data is contained within the article or supplementary material.

## Supplementary Materials

The following supporting information can be downloaded at: https://www.mdpi.com/article/10.3390/ma15093010/s1, Table S1: Properties and performance of magnetically recovered catalysts for sodium borohydride hydrolysis; Section: The synthesis of the catalysts from Figure 1; Table S2: Composition of the replacement solution used for the synthesis of cobalt catalysts from Figure 1 and corresponding reaction time; Figure S1: XRD pattern of the non-magnetic product of the galvanic replacement reaction in the synthesis of Co(Al) and bar chart of the XRD peaks for Al(acac)_3_ (PDF card 42-1746); Figure S2: The effect of the time of the ultrasonic treatment and the volume of the reaction mixture during the synthesis of Co(Al) catalysts on their activity in the hydrolysis of NaBH_4_. T = 40 °C. The reaction mixture contains 0.23 M ethanol solution of Co(acac)_3_ and 1 M aqueous solution of HCl. The Co:Al:HCl molar ratio is 0.2:1:0.27. Kinetic curves of hydrogen evolution over time (a) and hydrogen-generation rate (b) for each catalyst preparation condition; Figure S3: The effect of the Co:Al molar ratio in the reaction mixture during the synthesis of the Co(Al) catalysts on their activity in the hydrolysis of NaBH_4_. T = 40 °C. Kinetic curves of hydrogen evolution over time (a) and hydrogen-generation rate (b) for each molar ratio; Figure S4: The effect of the stirring modes on the hydrogen generation during NaBH_4_ hydrolysis in the presence of the Co(Al) catalyst. T = 40 °C. The set speed of the magnetic stirrer was 800 rpm. Kinetic curves of hydrogen evolution over time (a) and hydrogen-generation rate (b) for each stirring mode; Section: Kinetic calculations; Figure S5: (a) The effect of reaction temperature on hydrogen generation during NaBH_4_ hydrolysis in the presence of the Co(Al)NaOH catalyst. (b) The Arrhenius plot for NaBH_4_ hydrolysis on the Co(Al)NaOH catalyst; Table S3: The kinetic parameters of the hydrolysis of NaBH_4_ in the presence of the Co(Al)NaOH catalyst as calculated using the Langmuir–Hinshelwood model; Table S4: Elemental analysis data on the content of Co, Pt, and B in Co(Al)NaOH, Pt_0.2_Co_99.8_, Pt_2.5_Co_97.5_, and Pt_19_Co_81_ samples: Initial and after testing in 10 cycles of the NaBH_4_ hydrolysis without and with water washing; Figure S6: FTIR spectra of the Co(Al)NaOH catalyst: Initial and after testing in 10 cycles of NaBH_4_ hydrolysis without water washing; Figure S7: SEM images of the Pt_2.5_Co_97.5_ catalyst: (a) Initial, (b) tested in 10 cycles of NaBH_4_ hydrolysis, washed, and evacuated. References [83,84,85,86] are cited in the supplementary materials.
