# Supplementary material for: Magnetically Recovered Co and Co@Pt Catalysts Prepared by Galvanic Replacement on Aluminum Powder for Hydrolysis of Sodium Borohydride
Source: Materials (Basel). 2022 Apr 21;15(9):3010. doi: 10.3390/ma15093010 (PMC9103126; doi:10.3390/ma15093010)
Supplement: Supplementary file 1 [file materials-15-03010-s001.zip › materials-1645261-supplementary.pdf]

Supplementary Materials

Magnetically recovered Co and Co@Pt catalysts prepared by galvanic replacement on aluminum powder for hydrolysis of sodium borohydride

Anna M. Ozerova *, Anastasia A. Skobelkina, Valentina I. Simagina, Oksana V. Komova *, Igor P. Prosvirin, Olga A. Bulavchenko, Inna L. Lipatnikova, and Olga V. Netskina

Boreskov Institute of Catalysis, Pr. Akademika Lavrentieva 5, Novosibirsk 630090, Russia

*Correspondence: ozerova@catalysis.ru (A.M. Ozerova); komova@catalysis.ru (O.V. Komova)

Table S1. Properties and performance of magnetically recovered catalysts for sodium borohydride hydrolysis.

Catalyst	Catalyst description	Conditions	Activation energy, $kJ \cdot mol^{-1}$	Reaction rate, $mL \cdot min^{-1} \cdot g_{cat}^{-1}$	% of Initial Activity	Ref.	Ref. in manu-script
Cryogel p(APTMACl)*-Co	13.9 wt% Co NPs (10–50 nm) loaded into cationic cryogel	0.1 g catalyst; 50 ml solution; 50 mM $NaBH_4$; 5 wt% NaOH	37.36	250 (30 °C)	79.3% at 5th cycle	[1]	[31]
Co/IR-120	69.4 wt% Co (300–500 nm) deposited on Amberlite IR-120 resin beads (600 μm)	0.2 g catalyst; 100 ml solution; 5 wt% $NaBH_4$; 5 wt% NaOH	66.67	200 (without T control)	53% at 4th cycle	[2]	[32]
Co/SiO_2	46 wt% Co supported on porous spherical SiO_2	30 mg catalyst; 20 ml solution; 5 wt% $NaBH_4$; 5 wt% NaOH	59±2	8701 $mL \cdot min^{-1} \cdot g_{Co}^{-1}$ (40 °C)	–	[3]	[33]
Co@g-C_3N_4-rGO	Spheres of 78.1 wt% Co NPs (10–15 nm) core and g-C_3N_4 shell (5–10 nm) well distributed on reduced graphene oxide nanosheets	*Batch reactor* 20 mg catalyst; 20 ml solution; 0.1 M $NaBH_4$; 5 wt% NaOH	35.42	2565 (30 °C)	≈25% at 6th cycle	[4]	[34]
		Slurry-bed reactor 20 mg catalyst; 60 ml 0.2 M $NaBH_4$; 5 wt% NaOH	–	Constant 1844 $mL \cdot min^{-1} \cdot g_{cat}^{-1}$ for 43 min			
Pd/C-dots@ Fe_3O_4	Fe_3O_4 nanocubes covered with continuous carbon dots layers with Pd NPs (11.2 wt%)	1 mg catalyst; 5 ml solution; 2 wt% $NaBH_4$; 0.2 wt% NaOH	46.72	4890 (30 °C)	≈73% at 6th cycle	[5]	[35]
Co/Fe_3O_4@C	Fe_3O_4 spheres core (200–500 nm), carbon shell (35 nm) with Co NPs	40 mg catalyst; 50 ml solution; 158 mM $NaBH_4$	49.2	1403 (25 °C)	59% at 5th cycle	[6]	[36]
Fe_3O_4@C-Co	Hollow Fe_3O_4 spheres core (200–500 nm), carbon shell (12 nm) with Co NPs (3.46 wt%)	30 mg catalyst; 50 ml solution; 0.26 M $NaBH_4$	47.3	1746 (25 °C)	< 50% at 5th cycle	[7]	[37]
Co-Ni/ MWAC	Fe_3O_4 (21.2 nm) core, shell of walnut-based activated carbon with 8 wt% Co-Ni NPs (19.4 nm)	0.1 g catalyst; 5 wt% $NaBH_4$; 5 wt% NaOH	53.06	111 (30 °C)	–	[8]	[38]
Co-Ni/MSAS	Fe_3O_4 (34 nm) core, shell of Scharlau activated carbon with 8 wt% Co-Ni NPs (15.1 nm)		63.27	114 (30 °C)	–		

Catalyst	Catalyst description	Conditions	Activation energy, $kJ\cdot mol^{-1}$	Reaction rate, $mL\cdot min^{-1}\cdot g_{cat}^{-1}$	% of Initial Activity	Ref.	Ref. in manu-script
Co/Ni/MB	Fe_3O_4 (12.1 nm) core, shell of bentonite with 8 wt% Co-Ni NPs (9.7 nm)		44.98	186 (30 °C)	90% at 6th cycle		
Co/Ni/MZ	Fe_3O_4 (24.2 nm) core, shell of clinoptiolite zeolite with 8 wt% Co-Ni NPs (19.4 nm)		37.62	144 (30 °C)	–		
Co/Ni/ MWAC	Fe_3O_4 spheres core (< 30 nm), shell of activated carbon with Co (5.89 wt%) and Ni (1.91 wt%) NPs	0.1 g catalyst; 5 wt% $NaBH_4$; 5 wt% NaOH	40.7	740.7 (30 °C)	58% at 6th cycle	[9]	[39]
Co-Fe_3O_4-CNT	Fe_3O_4 NPs (20–25 nm) supported on multiwalled carbon nanotubes and impregnated with 5 wt% Co	0.06 wt% catalyst (0.03 g); 1 wt% $NaBH_4$ (0.5 g)	42.79	1213 (room T)	81% at 5th cycle, 65% at 8th cycle	[10]	[40]
CuBTC@ MAC	Cu or Cu and Co (metal content, 5.5 wt%) based metal organic frameworks supported on Fe_3O_4-magnetic activated carbon (< 30 nm)	0.01 g catalyst; 5 wt% $NaBH_4$; 5 wt% NaOH	10.3	5800 (30 °C)	66% at 10th cycle	[11]	[41]
CuCoBTC@ MAC			6.5	7900 (30 °C)	81% at 10th cycle		
Fe_2O_3@ OMWCNTs	Fe_2O_3 NPs (30–40 nm) supported on functionalized carbon nanotubes	37 mg catalyst; 50 ml solution; 0.5 g $NaBH_4$	15.92	264 (room T)	83% at 5th cycle	[12]	[42]
$CuFe_2O_4$/ RGO	Fusiform $CuFe_2O_4$ NPs (≈130 nm) supported on reduced graphene oxide	30 mg catalyst; 20 ml solution; 40 mg $NaBH_4$	33.95	622 (30 °C)	≈85% at 6th cycle	[13]	[43]
NiB/$NiFe_2O_4$	Spherical $NiFe_2O_4$ NPs (30–40 nm) with supported NiB (10 wt%)	0.1 g catalyst; 50 g water; 0.25 g $NaBH_4$; 0.5 g NaOH	72.52	299.88 (25 °C)	83.3% at 5th cycle	[14]	[44]
Fe@Co	Spherical particles (50–100 nm) with Fe core and Co shell. Co/(Co+Fe)=0.66 mol	10 mg catalyst; 10 ml solution; 5 wt% $NaBH_4$	35.62	–	–	[15]	[46]

* poly(3-acrylamidoproply)trimethylammonium chloride

The synthesis of the catalysts from Figure 1

The following commercial reagents were used as received: aluminum powder—ASD-0 grade (TU 1791–007–49421776–2011, Sual-PM); cobalt sulfate heptahydrate, $CoSO_4 \cdot 7H_2O$pure (GOST 4462-78, Vecton); cobalt chloride hexahydrate, $CoCl_2 \cdot 6H_2O$—analytically pure (GOST 4525-77, Ecros); ammonium chloride, NH_4Cl—analytically pure (GOST 3773-72, Reakhim); sodium citrate 5.5-hydrate, $Na_3C_6H_5O_7 \cdot 5.5H_2O$—analytically pure (GOST 22280-76, Ecros); acetylacetone, $C_5H_8O_2 \cdot 5H_2O$—analytically pure (GOST 10259-62, Reakhim); acetone, CH_3COCH_3—analytically pure (GOST 2603-79, Baza №1 Khimreactivov); hydrochloric acid, HCl—special purity 20-4 (GOST 14261-77, Sigma Tek); sodium hydroxide, NaOH—pure (GOST 4328-77, Reakhim); chloroplatinic (IV) acid hexahydrate, $H_2PtCl_6 \cdot 6H_2O$—38.01 wt% of Pt (TU 2612-034-00205067-2003, Aurat); sodium borohydride, $NaBH_4$—purity of 98 wt% (CAS 16940-66-2, Chemical Line).

Cobalt catalysts from Figure 1 were synthesized by the galvanic replacement reaction using Al particles as a template by varying the composition of the replacement solution (Table S2). First, 0.5 g of Al powder was degreased in acetone and etched in 1 M HCl solution (5 mL) for 10 min to remove the surface oxide layer. Then 15 mL of the replacement solution was added to the Al suspension. The Co:Al molar ratio was 0.2:1. The reaction was carried out in an ultrasonic bath (Sapfir, Russia) at 60 °C and 100 W for 2 hours. The time of reaction depended on the composition of the replacement solution (Table S2). After the completion of the reaction (stopping the formation of gas bubbles), the resulting sample was separated from the reaction medium with a magnet, washed 5 times with distilled water and 3 times with acetone, and evacuated for 2 h at room temperature.

Table S2. Composition of the replacement solution used for the synthesis of cobalt catalysts from Figure 1 and corresponding reaction time.

**Composition of the replacement solution**	**Time of reaction**
15 mL of water solution containing 0.23 M $CoSO_4{\cdot}7H_2O$ (0.82 g), 1.87 M NH_4Cl (1.5 g), 0.44 M $Na_3C_6H_5O_7{\cdot}5.5H_2O$ (1.7 g)	3.5 h
15 mL of water solution containing 0.23 M $CoCl_2{\cdot}6H_2O$ (0.82 g), 1.87 M NH_4Cl (1.5 g), 0.44 M $Na_3C_6H_5O_7{\cdot}5.5H_2O$ (1.7 g)	3.5 h
15 mL of water solution containing 0.23 M $CoCl_2{\cdot}6H_2O$ (0.82 g)	2 h
15 mL of ethanol solution containing 0.23 M $CoCl_2{\cdot}6H_2O$ (0.82 g)	1 h
Mixture containing 13 mL water, 0.82 g $CoCl_2{\cdot}6H_2O$, 2 mL acetylacetone	2 h
15 mL of ethanol solution containing 0.23 M $C_{10}H_{14}O_4Co{\cdot}2H_2O$ (1.0115 g)	2.5 h
15 mL of ethanol solution containing 0.23 M $C_{15}H_{21}O_6Co$ (1.2278 g)	2 h

Cobalt (II) acetylacetonate dihydrate was prepared as reported elsewhere [16]. Acetylacenote (4.5 mL, 0.044 mol) was added slowly with stirring to a 15 mL aqueous solution of NaOH (1.6 g, 0.04 mol). The resulting yellow solution was added drop by drop with vigorous stirring to a 25 mL aqueous solution of $CoCl_2{\cdot}6H_2O$ (4.8 g, 0.02 mol). The precipitate formed was filtered, washed with water, and recrystallized from a hot mixture of ethanol and chloroform. The orange crystals were filtered, washed in ethanol, and air dried.

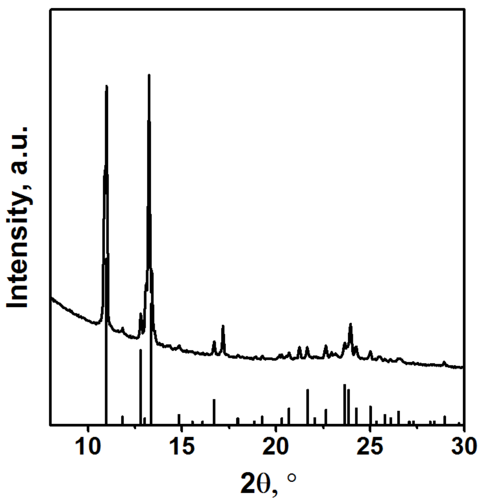

Figure S1. XRD pattern of the non-magnetic product of the galvanic replacement reaction in the synthesis of Co(Al) and bar chart of the XRD peaks for $Al(acac)_3$ (PDF card 42-1746).

(a)

(b)

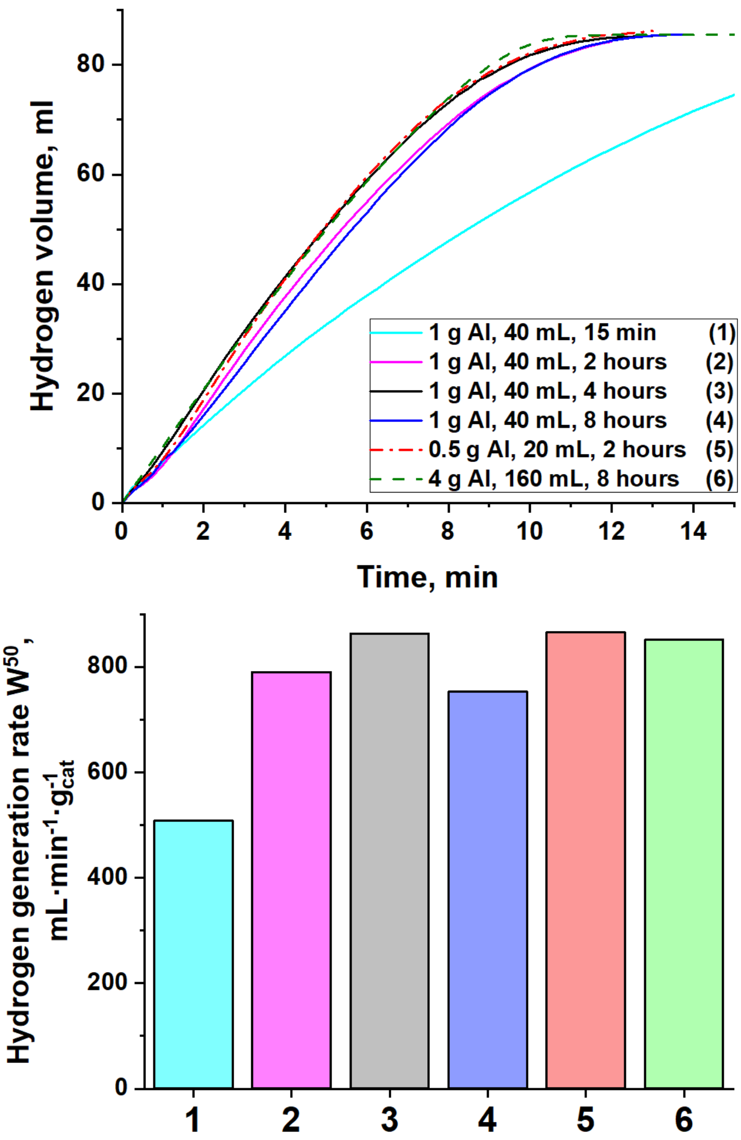

Figure S2. The effect of the time of the ultrasonic treatment and the volume of the reaction mixture during the synthesis of Co(Al) catalysts on their activity in the hydrolysis of $NaBH_4$. T = 40 °C. The reaction mixture contains 0.23 M ethanol solution of $Co(acac)_3$ and 1 M aqueous solution of HCl. The Co:Al:HCl molar ratio is 0.2:1:0.27. Kinetic curves of hydrogen evolution over time (**a**) and hydrogen-generation rate (**b**) for each catalyst preparation condition.

(a)

(b)

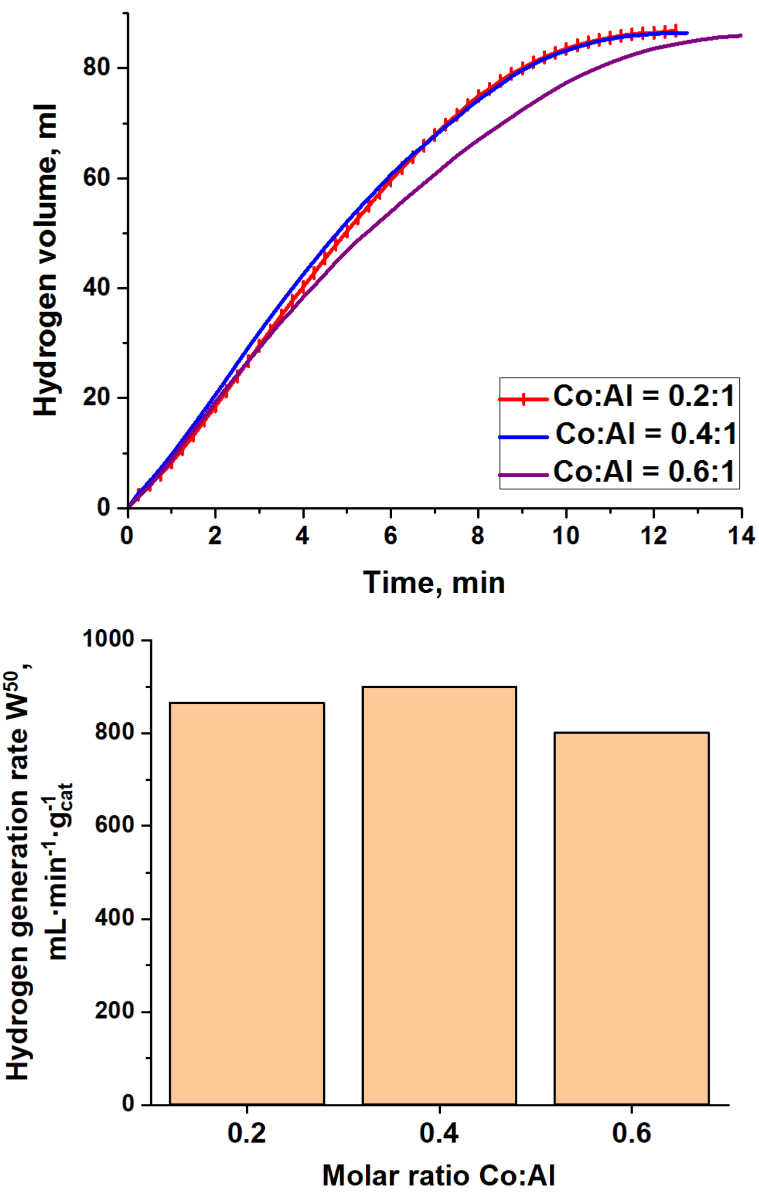

Figure S3. The effect of the Co:Al molar ratio in the reaction mixture during the synthesis of the Co(Al) catalysts on their activity in the hydrolysis of $NaBH_4$. T = 40 °C. Kinetic curves of hydrogen evolution over time (**a**) and hydrogen-generation rate (**b**) for each molar ratio.

(a)

(b)

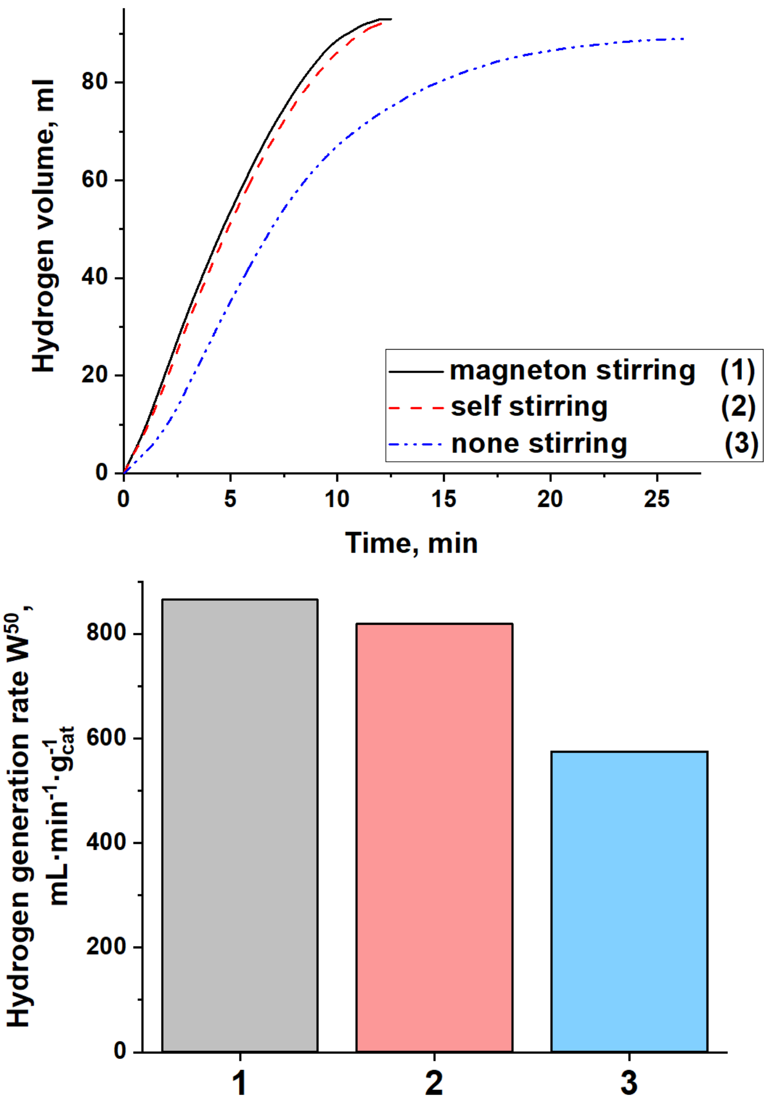

Figure S4. The effect of the stirring modes on the hydrogen generation during $NaBH_4$ hydrolysis in the presence of the Co(Al) catalyst. T = 40 °C. The set speed of the magnetic stirrer was 800 rpm. Kinetic curves of hydrogen evolution over time (**a**) and hydrogen-generation rate (**b**) for each stirring mode.

Kinetic calculations

According to the literature [17–19], the kinetics of the hydrolysis of $NaBH_4$ in the presence of heterogeneous catalysts is described by the Langmuir–Hinshelwood model. In this case, the following expression is used for the approximation of experimental data:

$$(C_0 - C_t) + \frac{1}{K_a} \cdot \ln\left(\frac{C_0}{C_t}\right) = kt, \qquad \text{(S1)}$$

where C_0 (mol·L^{-1}) is the initial concentration of $NaBH_4$, C_t (mol·L^{-1}) is its current concentration, K_a (L·mol^{-1}) is the adsorption constant of $NaBH_4$ and k (mol·L^{-1}·s^{-1}) is the rate constant of the catalytic hydrolysis of $NaBH_4$.

The reaction rate constant (k) is expressed according to the Arrhenius equation as

$$k = k_0 \cdot \exp\left(-\frac{E_a}{RT}\right), \qquad \text{(S2)}$$

where E_a is the activation energy of the process.

Therefore, experimental data from $NaBH_4$ hydrolysis in the presence of the Co(Al)NaOH catalyst at temperatures ranging from 20 to 40 °C (Figure S5a) were analyzed using the Langmuir–Hinshelwood model, and the results are presented in Table S3.

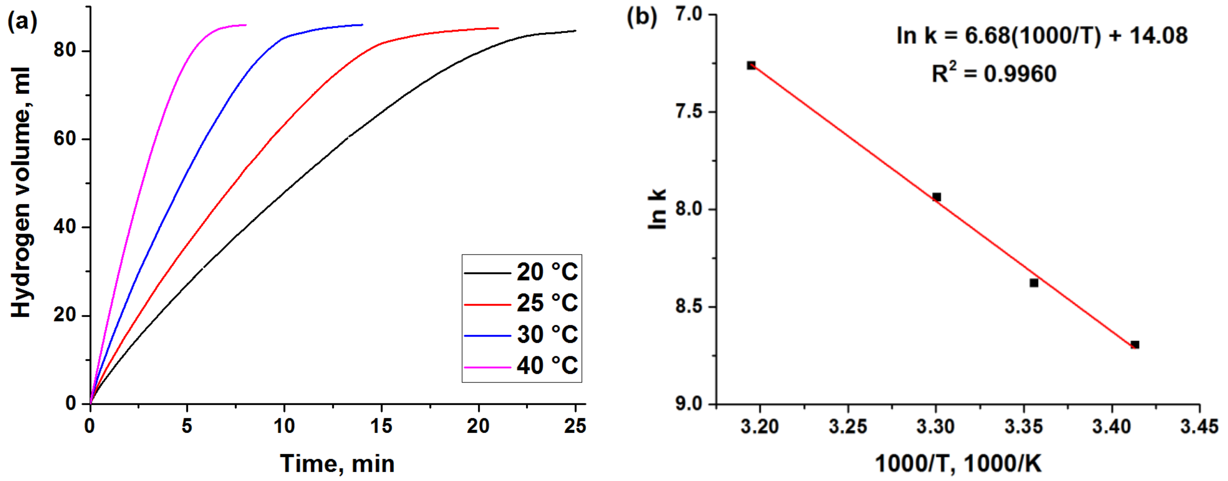

Figure S5. (**a**) The effect of reaction temperature on hydrogen generation during $NaBH_4$ hydrolysis in the presence of the Co(Al)NaOH catalyst. (**b**) The Arrhenius plot for $NaBH_4$ hydrolysis on the Co(Al)NaOH catalyst.

Table S3. The kinetic parameters of the hydrolysis of $NaBH_4$ in the presence of the Co(Al)NaOH catalyst as calculated using the Langmuir–Hinshelwood model.

**Temperature, K**	**Adsorption constant (K_a), L/mol**	**R^2**	**Reaction rate constant (k), mol·L^{-1}·min^{-1}**
293	15.5	0.9995	0.0101±0.0001
298	16.5	0.9997	0.0139±0.0001
308	17	0.9998	0.0214±0.0001
313	13.5	0.9997	0.0423±0.0001

The reaction rate constant (k) at different temperatures was plotted on an Arrhenius plot (Figure S5b), and the activation energy of the catalyzed reaction was calculated. It was found to be 55.5±1.9 kJ/mol.

Table S4. Elemental analysis data on the content of Co, Pt, and B in Co(Al)NaOH, $Pt_{0.2}Co_{99.8}$, $Pt_{2.5}Co_{97.5}$, and $Pt_{19}Co_{81}$ samples: Initial and after testing in 10 cycles of the $NaBH_4$ hydrolysis without and with water washing.

Sample	Total	Co		Pt		B	
	g	wt%	g	wt%	g	wt%	g
**Co(Al)NaOH**							
Initial	1	98.1	0.9810				
Tested	1.8597	56.7	1.0541			5.85	0.1090
Tested, after washing	0.9793	99.3	0.9729			1.08	0.0107
**$Pt_{0.2}Co_{99.8}$**							
Initial	1	94.3	0.9430	0.59	0.0057		
Tested, after washing	0.9523	96.8	0.9217	0.67	0.0064*	0.96	0.0093
**$Pt_{2.5}Co_{97.5}$**							
Initial	1	91.0	0.9100	7.82	0.0782		
Tested	1.3704	76.8	1.0527	4.6	0.0634	0.98	0.0135
Tested, after washing	0.9423	93.5	0.8811	6.06	0.0570	1.17	0.0107
**$Pt_{19}Co_{81}$**							
Initial	1	57.5	0.5750	43.7	0.4370		
Tested	1.4509	48.8	0.7080	35.8	0.5192	2.45	0.0356
Tested, after washing	0.9736	56.2	0.5470	39.0	0.3796	0.52	0.0050

* An increased Pt content is due to the measurement error specified in Part 2.3.

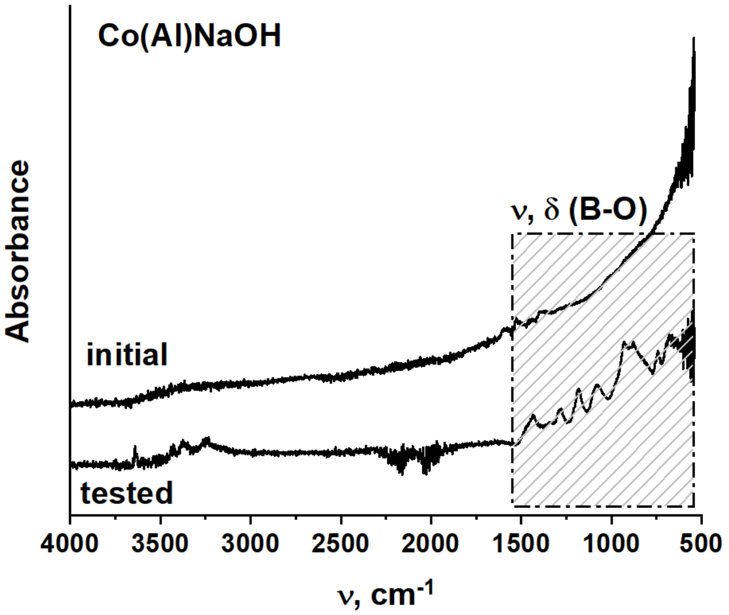

Figure S6. FTIR spectra of the Co(Al)NaOH catalyst: Initial and after testing in 10 cycles of $NaBH_4$ hydrolysis without water washing.

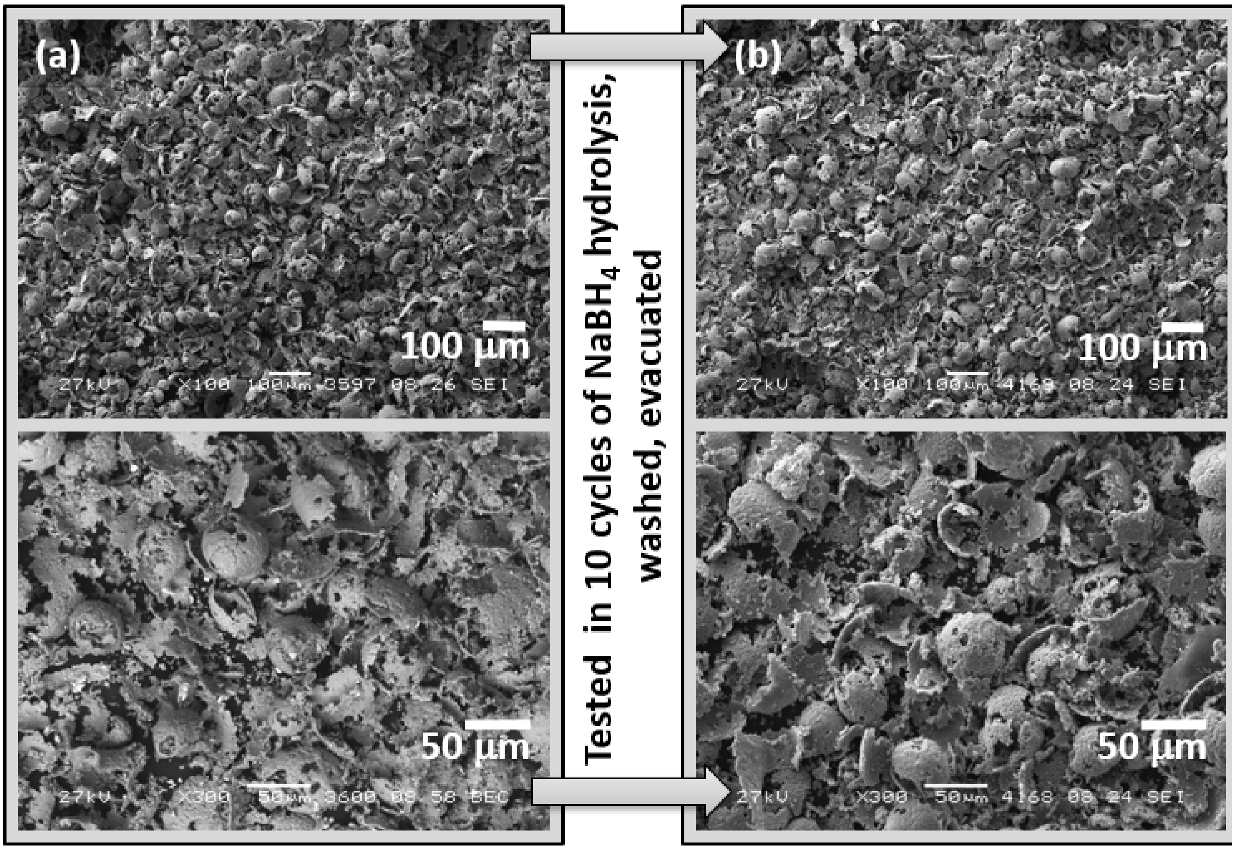

Figure S7. SEM images of the $Pt_{2.5}Co_{97.5}$ catalyst: (**a**) Initial, (**b**) tested in 10 cycles of $NaBH_4$ hydrolysis, washed, and evacuated.

References

1. Sahiner, N.; Seven, F. A facile synthesis route to improve the catalytic activity of inherently cationic and magnetic catalyst systems for hydrogen generation from sodium borohydride hydrolysis. *Fuel Process. Technol.* **2015**, *132*, 1–8, doi:10.1016/j.fuproc.2014.12.008.

2. Liu, C.H.; Chen, B.H.; Hsueh, C.L.; Ku, J.R.; Tsau, F.; Hwang, K.J. Preparation of magnetic cobalt-based catalyst for hydrogen generation from alkaline $NaBH_4$ solution. *Appl. Catal. B Environ.* **2009**, *91*, 368–379, doi:10.1016/j.apcatb.2009.06.003.

3. Shih, Y.J.; Su, C.C.; Huang, Y.H.; Lu, M.C. SiO_2-supported ferromagnetic catalysts for hydrogen generation from alkaline NaBH4 (sodium borohydride) solution. *Energy* **2013**, *54*, 263–270, doi:10.1016/j.energy.2013.01.063.

4. Duan, S.; Han, G.; Su, Y.; Zhang, X.; Liu, Y.; Wu, X.; Li, B. Magnetic Co@g-C_3N_4 core-shells on rGO sheets for momentum transfer with catalytic activity toward continuous-flow hydrogen generation. *Langmuir* **2016**, *32*, 6272–6281, doi:10.1021/acs.langmuir.6b01248.

5. Guo, Y.; Qian, J.; Iqbal, A.; Zhang, L.; Liu, W.; Qin, W. Pd nanoparticles immobilized on magnetic carbon dots@Fe_3O_4 nanocubes as a synergistic catalyst for hydrogen generation. *Int. J. Hydrogen Energy* **2017**, *42*, 15167–15177, doi:10.1016/j.ijhydene.2017.04.253.

6. Chen, B.; Chen, S.; Bandal, H.A.; Appiah-Ntiamoah, R.; Jadhav, A.R.; Kim, H. Cobalt nanoparticles supported on magnetic core-shell structured carbon as a highly efficient catalyst for hydrogen generation from $NaBH_4$ hydrolysis. *Int. J. Hydrogen Energy* **2018**, *43*, 9296–9306, doi:10.1016/j.ijhydene.2018.03.193.

7. Baye, A.F.; Abebe, M.W.; Appiah-Ntiamoah, R.; Kim, H. Engineered iron-carbon-cobalt (Fe_3O_4@C-Co) core-shell composite with synergistic catalytic properties towards hydrogen generation via $NaBH_4$ hydrolysis. *J. Colloid Interface Sci.* **2019**, *543*, 273–284, doi:10.1016/j.jcis.2019.02.065.

8. Didehban, A.; Zabihi, M.; Babajani, N. Preparation of the efficient nano-bimetallic cobalt-nickel catalysts supported on the various magnetic substrates for hydrogen generation from hydrolysis of sodium borohydride in alkaline solutions. *Polyhedron* **2020**, *180*, 114405, doi:10.1016/j.poly.2020.114405.

9. Soltani, M.; Zabihi, M. Hydrogen generation by catalytic hydrolysis of sodium borohydride using the nano-bimetallic catalysts supported on the core-shell magnetic nanocomposite of activated carbon. *Int. J. Hydrogen Energy* **2020**, *45*, 12331–12346, doi:10.1016/j.ijhydene.2020.02.203.

10. Bandal, H.A.; Jadhav, A.R.; Kim, H. Cobalt impregnated magnetite-multiwalled carbon nanotube nanocomposite as magnetically separable efficient catalyst for hydrogen generation by $NaBH_4$ hydrolysis. *J. Alloys Compd.* **2017**, *699*, 1057–1067, doi:10.1016/j.jallcom.2016.12.428.

11. Faghihi, M.; Akbarbandari, F.; Zabihi, M.; Pazouki, M. Synthesis and characterization of the magnetic supported metal-organic framework catalysts (CuCoBTC@MAC and CuBTC@MAC) for the hydrogen production from sodium borohydride. *Mater. Chem.*

Phys. **2021**, *267*, 124599, doi:10.1016/j.matchemphys.2021.124599.

12. Prasad, D.; Patil, K.N.; Sandhya, N.; Chaitra, C.R.; Bhanushali, J.T.; Samal, A.K.; Keri, R.S.; Jadhav, A.H.; Nagaraja, B.M. Highly efficient hydrogen production by hydrolysis of $NaBH_4$ using eminently competent recyclable Fe2O3 decorated oxidized MWCNTs robust catalyst. *Appl. Surf. Sci.* **2019**, *489*, 538–551, doi:10.1016/j.apsusc.2019.06.041.
13. Tang, M.; Xia, F.; Gao, C.; Qiu, H. Preparation of magnetically recyclable $CuFe_2O_4$/RGO for catalytic hydrolysis of sodium borohydride. *Int. J. Hydrogen Energy* **2016**, *41*, 13058–13068, doi:10.1016/j.ijhydene.2016.05.034.
14. Liang, Z.; Li, Q.; Li, F.; Zhao, S.; Xia, X. Hydrogen generation from hydrolysis of $NaBH_4$ based on high stable NiB/$NiFe_2O_4$ catalyst. *Int. J. Hydrogen Energy* **2017**, *42*, 3971–3980, doi:10.1016/j.ijhydene.2016.10.115.
15. Tsai, C.W.; Chen, H.M.; Liu, R.S.; Lee, J.-F.; Chang, S.M.; Weng, B.J. Magnetically recyclable Fe@Co core-shell catalysts for dehydrogenation of sodium borohydride in fuel cells. *Int. J. Hydrogen Energy* **2012**, *37*, 3338–3343, doi:10.1016/j.ijhydene.2011.11.038.
16. Goff, H.M.; Hines, J.; Griesel, J.; Mossman, C. Synthesis, characterization, and use of a cobalt(II) complex as an NMR shift reagent: An integrated laboratory experiment. *J. Chem. Educ.* **1982**, *59*, 422–423, doi:10.1021/ed059p422.
17. Hung, A.J.; Tsai, S.F.; Hsu, Y.Y.; Ku, J.R.; Chen, Y.H.; Yu, C.C. Kinetics of sodium borohydride hydrolysis reaction for hydrogen generation. *Int. J. Hydrogen Energy* **2008**, *33*, 6205–6215, doi:10.1016/j.ijhydene.2008.07.109.
18. Andrieux, J.; Demirci, U.B.; Miele, P. Langmuir-Hinshelwood kinetic model to capture the cobalt nanoparticles-catalyzed hydrolysis of sodium borohydride over a wide temperature range. *Catal. Today* **2011**, *170*, 13–19, doi:10.1016/j.cattod.2011.01.019.
19. Retnamma, R.; Novais, A.Q.; Rangel, C.M. Kinetics of hydrolysis of sodium borohydride for hydrogen production in fuel cell applications: A review. *Int. J. Hydrogen Energy* **2011**, *36*, 9772–9790, doi:10.1016/j.ijhydene.2011.04.223.
